# Supplementary material for: Dynamic changes in the epigenomic landscape regulate human organogenesis and link to developmental disorders
Source: Nat Commun. 2020 Aug 6;11:3920. doi: 10.1038/s41467-020-17305-2 (PMC7413392; doi:10.1038/s41467-020-17305-2)
Supplement: Supplementary file 10 — Supplementary Information [file 41467_2020_17305_MOESM10_ESM.pdf]

Supplementary file for: Dynamic changes in the epigenomic landscape regulate human organogenesis and link to developmental disorders

Dave T. Gerrard, Andrew A. Berry, Rachel E. Jennings, Matthew J Birket, Peyman Zarrineh, Myles G. Garstang, Sarah L Withey, Patrick Short, Sandra Jiménez-Gancedo, Panos N Firbas, Ian Donaldson, Andrew D. Sharrocks, Karen Piper Hanley, Matthew E Hurles, José Luis Gomez-Skarmeta, Nicoletta Bobola and Neil A. Hanley

SUPPLEMENTARY DATA

Nine supplementary tables are available in the separate Supplementary Data file.

SUPPLEMENTARY FIGURES

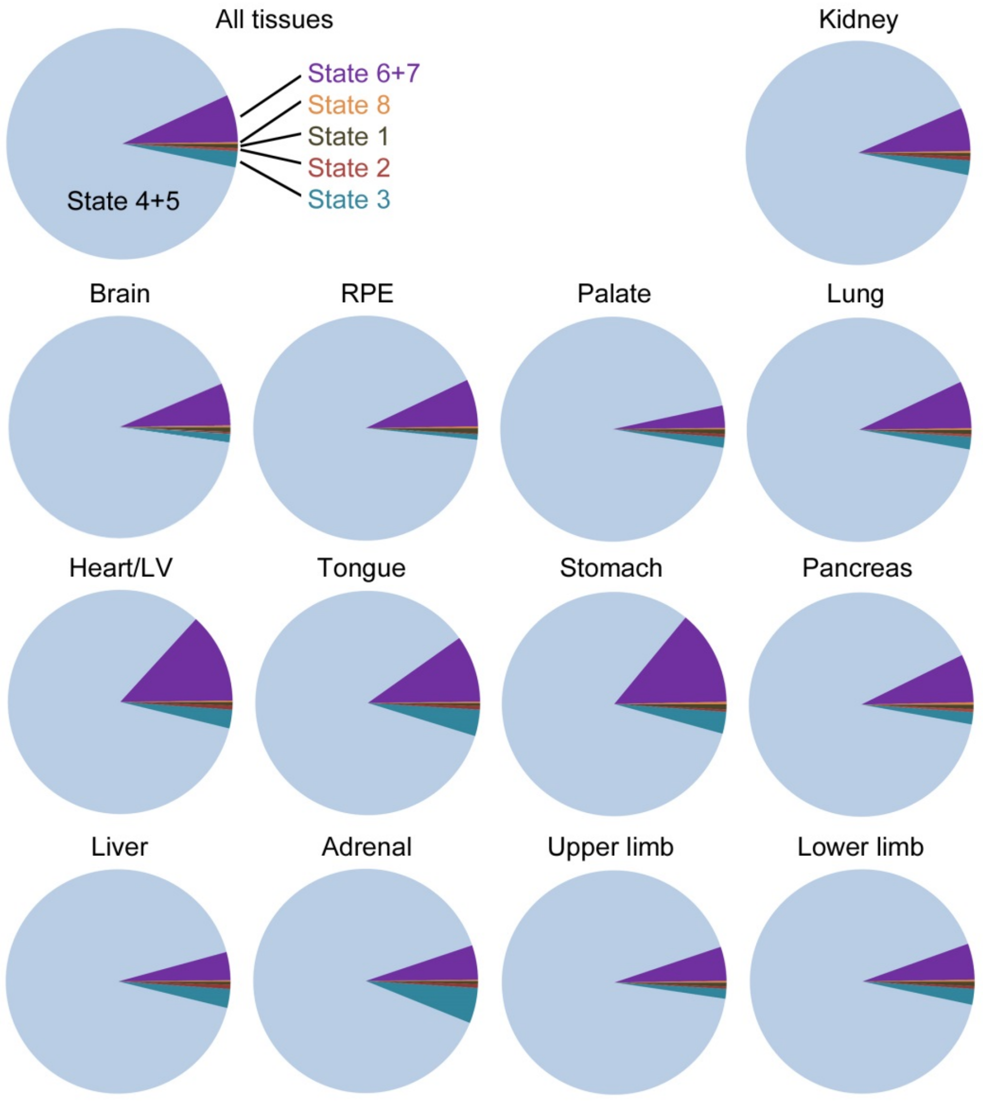

Supplementary figure 1. Genome coverage for the different histone modifications in all tissues.

This figure relates to Figure 1. Pie charts for individual tissues of genome coverage according to chromatin state by ChromHMM. The average for all tissues is shown in Figure 1c.

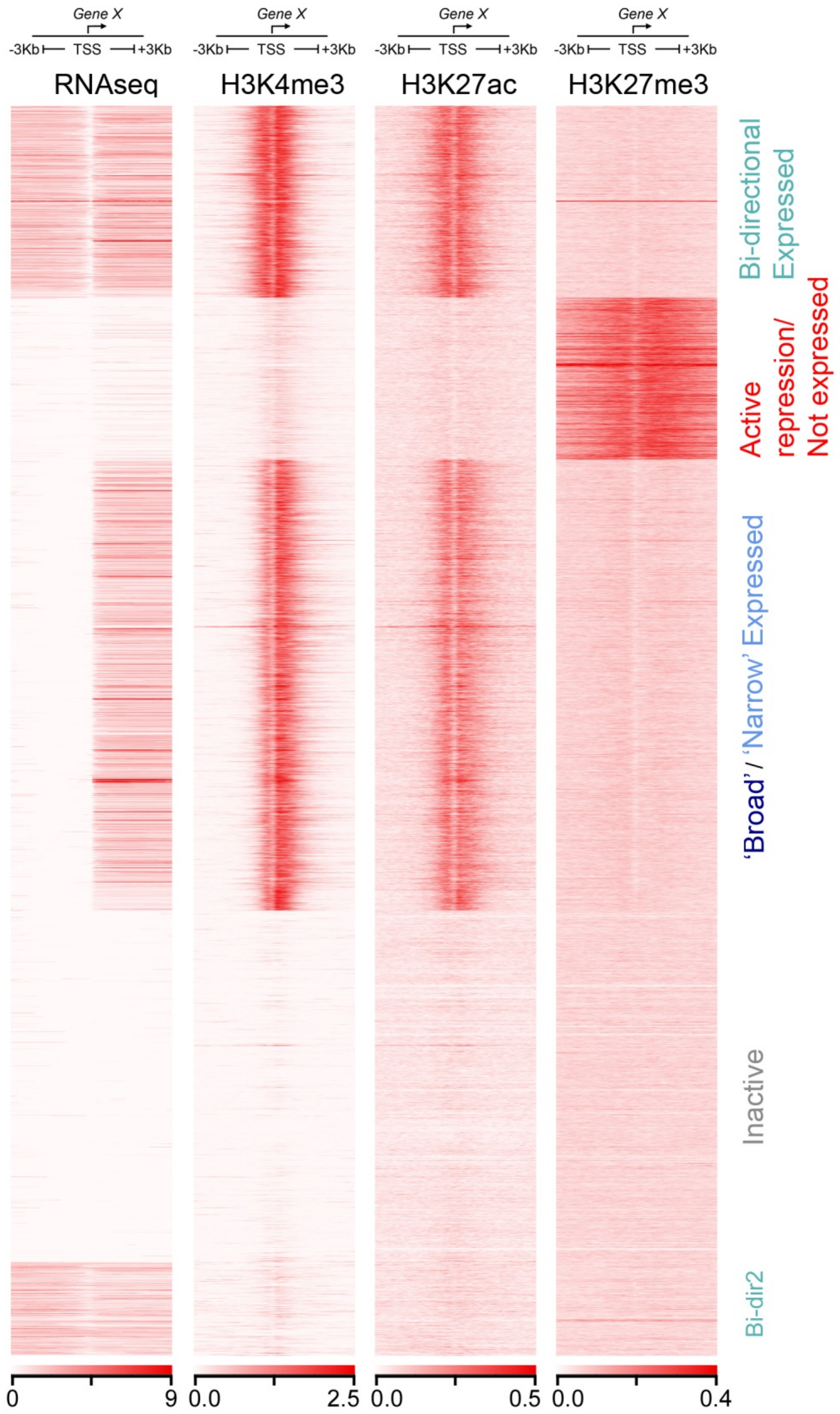

Supplementary figure 2. Variant promoter state observed for retinal pigmented epithelium (RPE).

This figure relates to Figure 2. For the major five promoter states, in RPE the Broad and Narrow expressed categories identified in most tissues and shown in Figure 2a were aggregated by ngsplot and termed Broad/narrow expressed. This occurred in favour and as a consequence of clustering a subset of genes with less robust bidirectional transcription and barely any marking for H3K4me3 or H3K27ac (termed Bi-dir2).

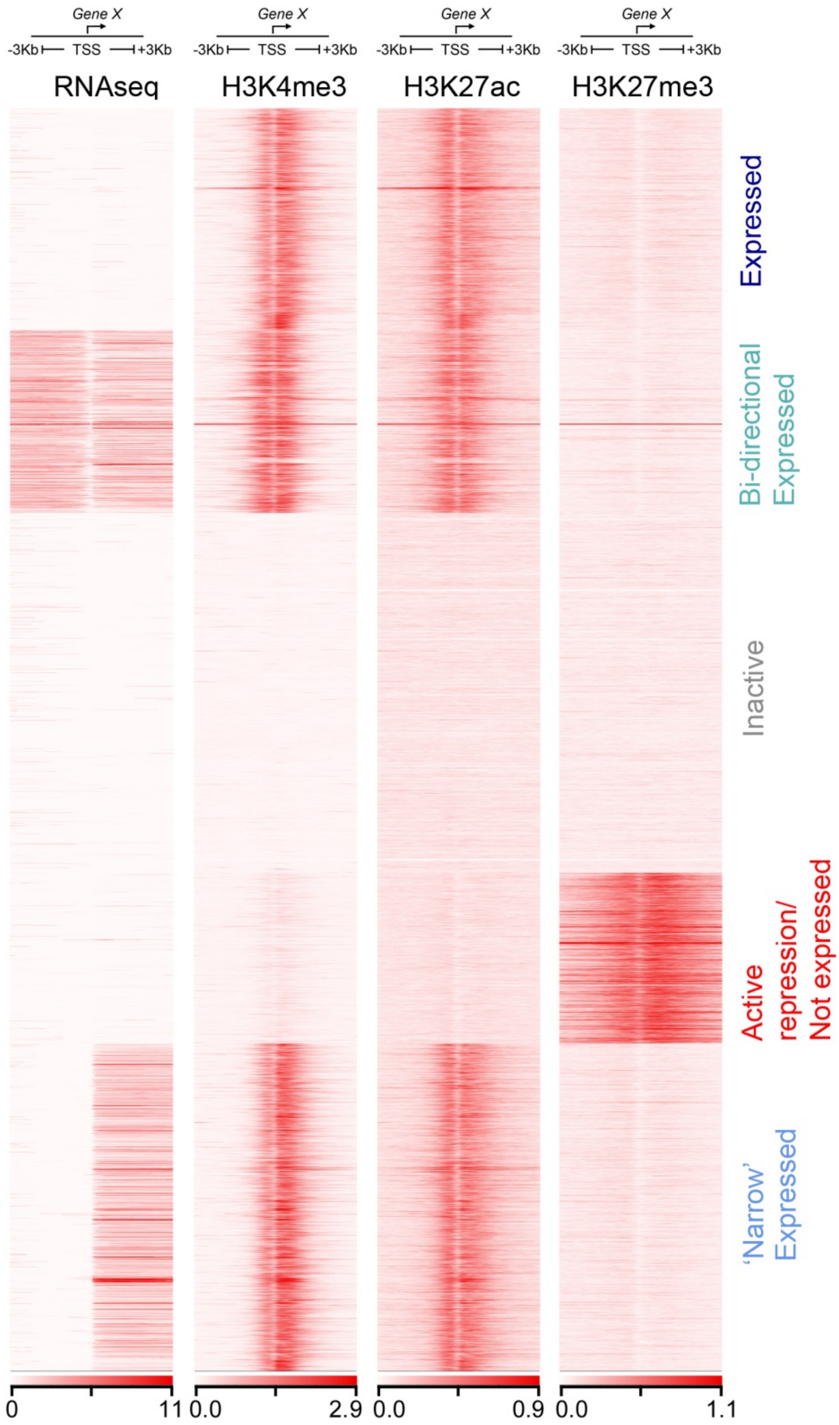

Supplementary figure 3. Variant promoter state observed for brain, lung and liver.

This figure relates to Figure 2. For the five major promoter states, in brain, lung and liver the Broad expressed category, evident in Figure 2a, is marked as Expressed (example shown for lung). While the H3K4me3 and H3K27ac marks were indistinguishable from the Broad expressed category of Figure 2a, there was no accompanying RNAseq detection at the TSS. These genes encoded longer transcripts with characteristically long first introns (Supplementary figure 4) leading to an under-representation of RNAseq reads at the TSS. Total transcript count across the entire gene was equivalent for Broad expressed (Figure 2a) and Expressed genes.

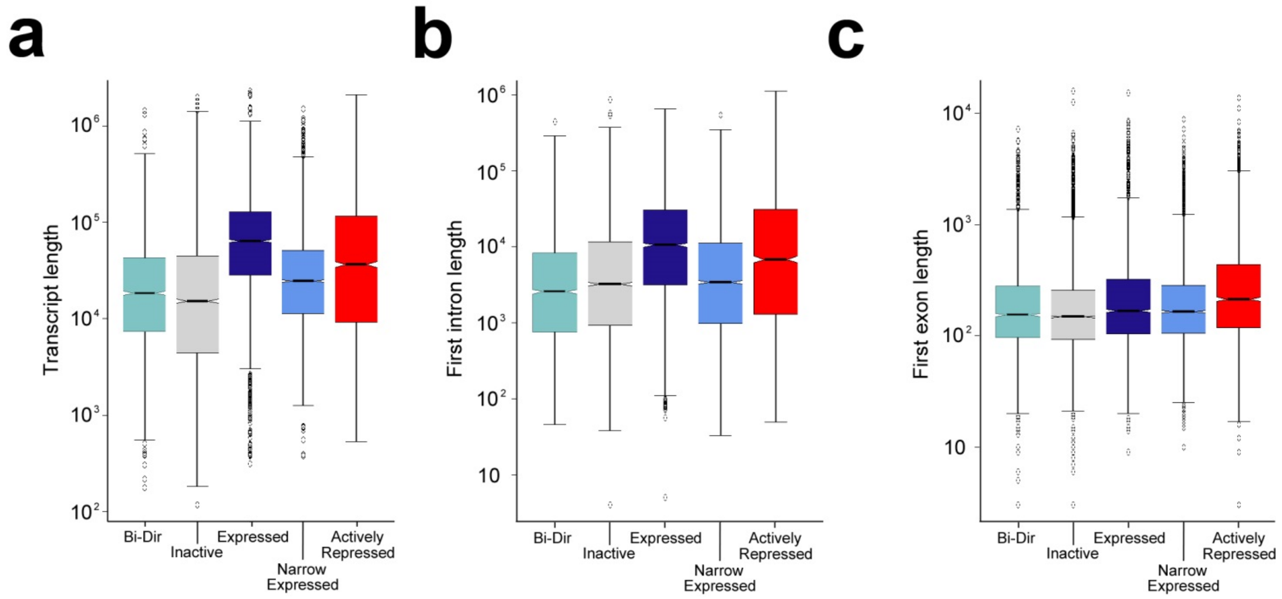

Supplementary figure 4. Characteristics of the Expressed promoter state.

This figure relates to Figure 2 and Supplementary figure 3. **a)** Transcript length **b)** first intron length and **c)** first exon length for the categories of genes detected in brain, lung and liver (Supplementary figure 3). Although overall transcript counts for Expressed were similar to Broad Expressed (Figure 2a), RNAseq was not detected at the TSS due to longer first introns and overall transcript length. The first exon was similar to other categories. The example shown is for lung with the same findings observed for brain and liver. The boxes around the median contain the inter-quartile range (IQR) between the upper (75%) and lower (25%) quartiles. The whiskers extend to 1.5xIQR and data-points outside of this are drawn individually.

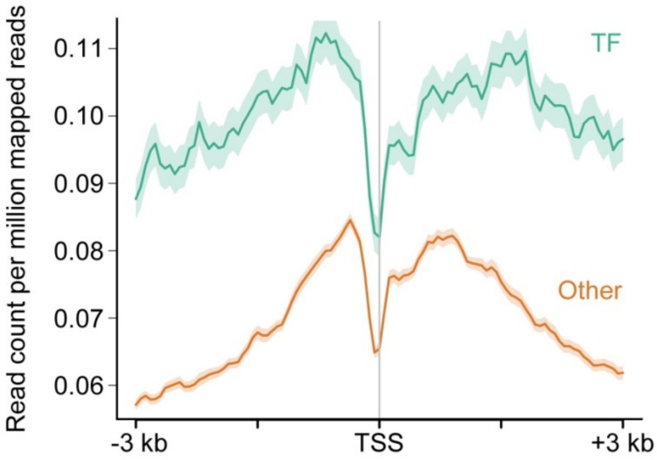

Supplementary figure 5. Levels of H3K27me3 at the TSS of actively repressed genes which encoded transcription factors (TF) compared to those encoding all other proteins.

This figure relates to Figure 2. Within the Active Repression category across all tissues genes encoding transcription factors (TFs) possessed appreciably greater marking with H3K27me3 at their transcriptional start site (TSS) compared to those genes encoding other proteins (Other).

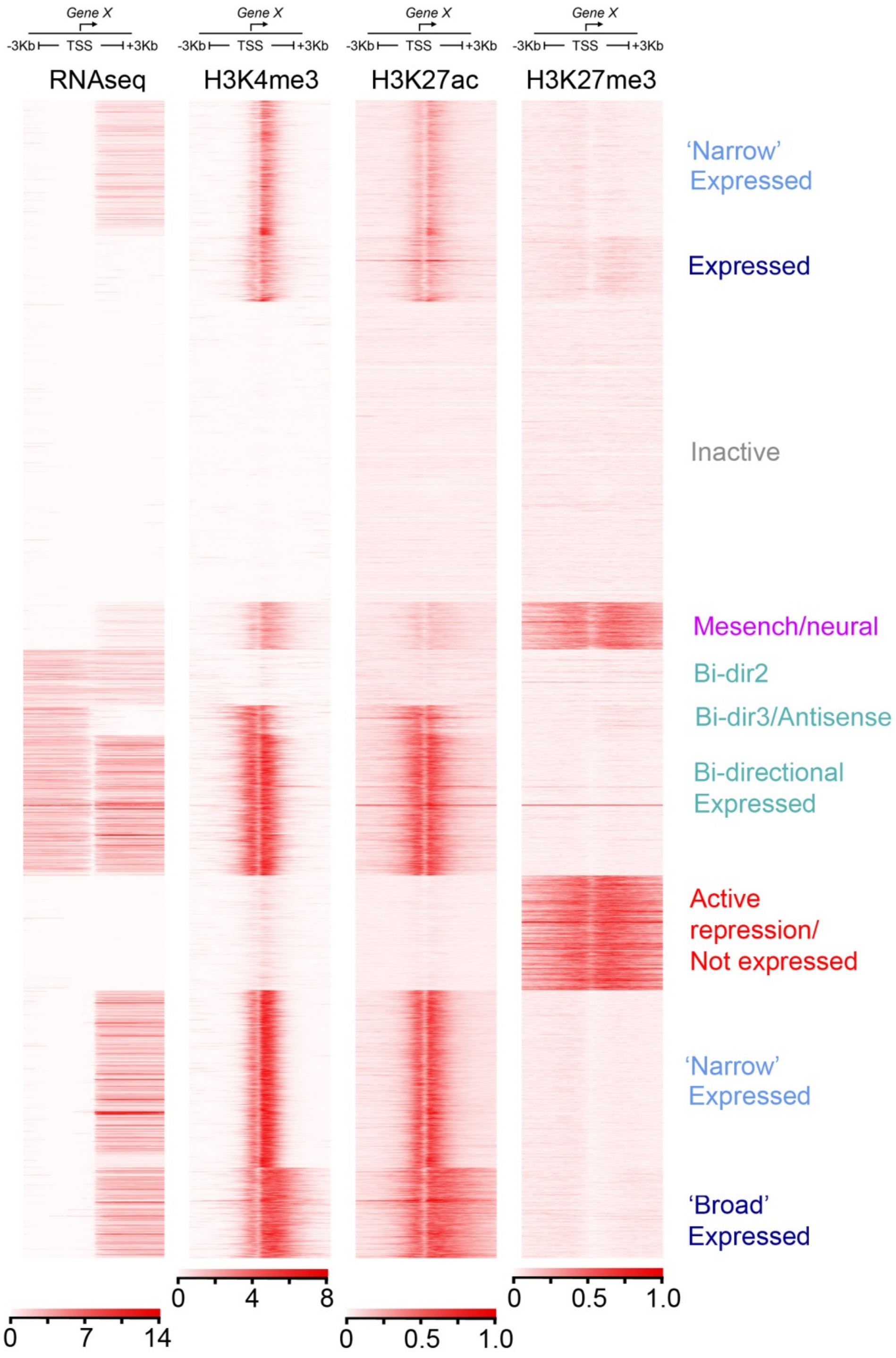

Supplementary figure 6. Extended categorisation of promoter states in adrenal to identify a combined H3K27me3 / H3K4me3 signal.

This example is for adrenal gland, which required extension of ngsplot parameters[1] to allow ten clusters in order to split the H3K27me3 signal and identify a sub-category which also contained H3K4me3 (as a by-product, this modification also identified the major promoter states identified in Supplementary figures 2 & 3 and additional sub-categories of bidirectional or antisense transcripts). Rather than bivalency, the genes underlying the combined H3K27me3 and H3K4me3 signal were characteristic of mesenchyme/vascular and nerves (Mesench/neural) (see the accompanying ReactomePA plot in Supplementary figure 7.

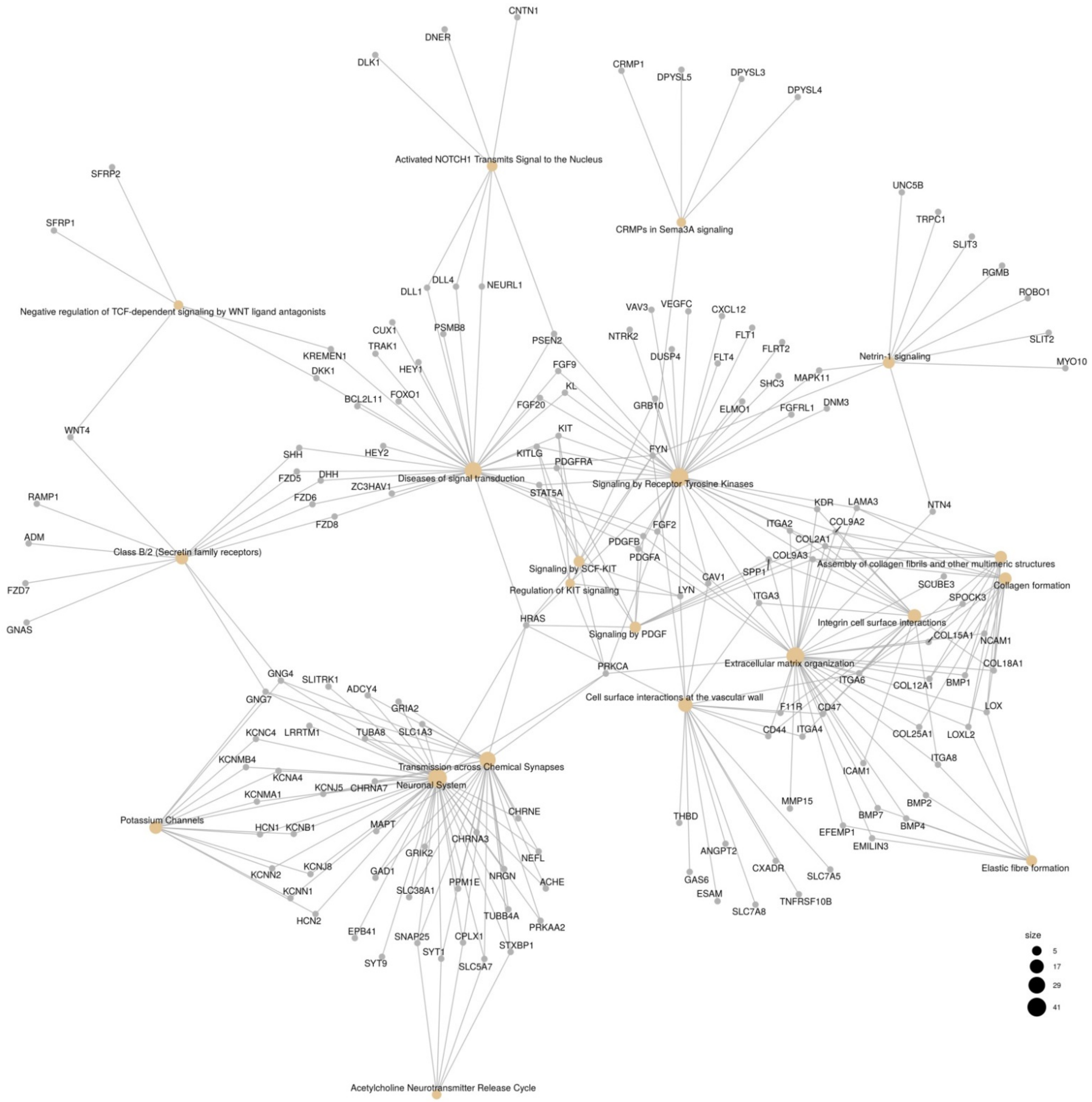

Supplementary figure 7. ReactomePA plot for genes underlying the dual H3K27me3 and H3K4me3 signal in adrenal gland.

This figure relates to Supplementary figure 6. The analysis shows gene set enrichment and functional assignment as part of ReactomePA[2]. The visualisation, which can be magnified, demonstrates the major functional annotations for the dual H3K27me3 and H3K4me3 signal in Supplementary figure 6 relate to common features of mesenchyme/vascular (e.g. extracellular matrix (ECM) organisation, integrins, regulation of KIT signalling, signal transduction and receptor tyrosine kinase signalling) and neural tissue (e.g. neuronal system, transmission across chemical synapses and netrin signalling). These findings were common to the sub-category of dual H3K27me3 and H3K4me3 signal in all tissues. Circle size reflects the number of connected genes.

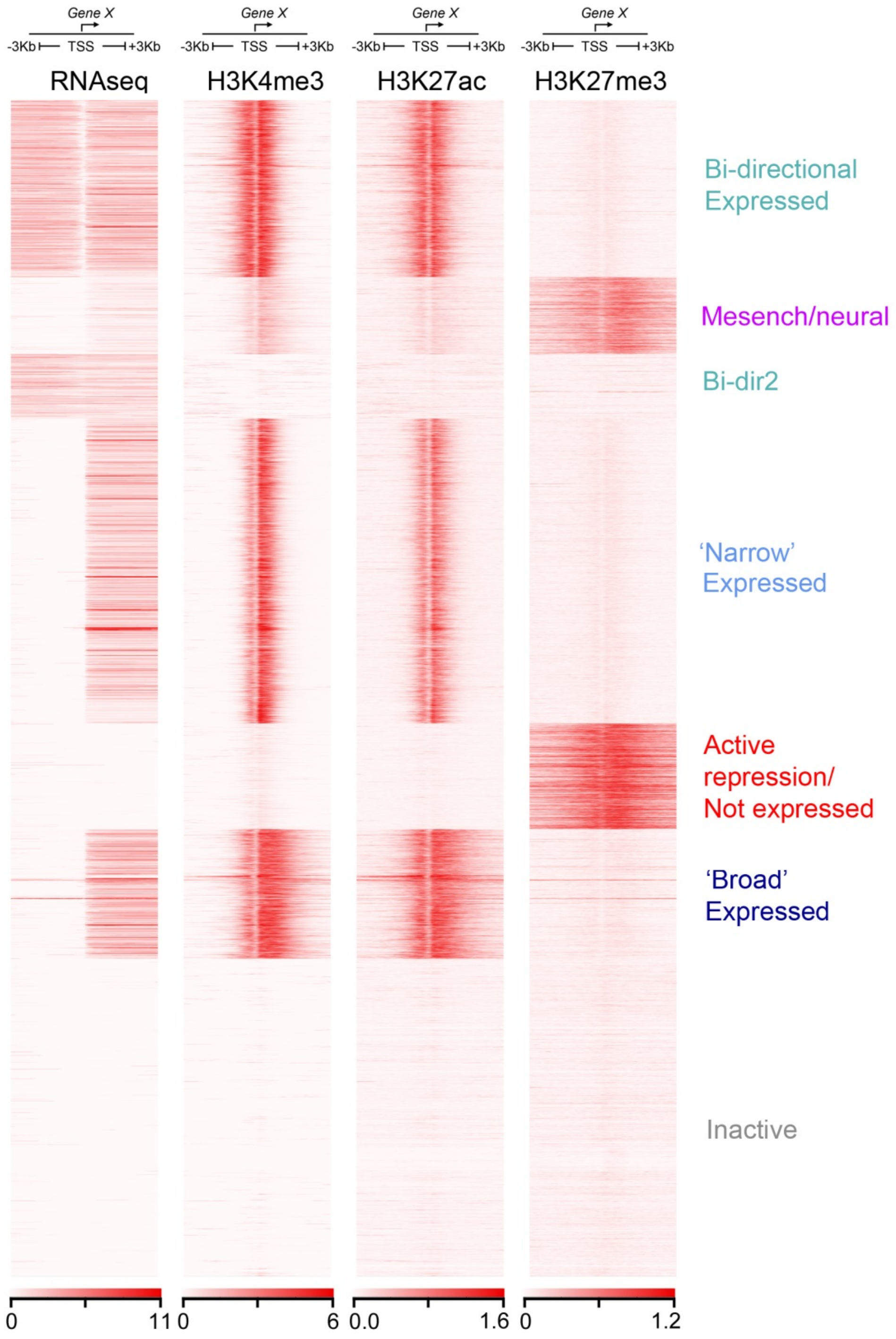

Supplementary figure 8. Extended categorisation of promoter states in kidney to identify a combined H3K27me3 / H3K4me3 signal.

This example is for kidney, which required extension of ngsplot parameters[1] to allow seven clusters in order to split the H3K27me3 signal and identify a sub-category which also contained H3K4me3 (as a by-product, this modification also identified the major promoter state, Bi-dir2, identified in Supplementary figures 2). Rather than bivalency, the genes underlying the combined H3K27me3 and H3K4me3 signal were characteristic of mesenchyme/vascular and nerves (Mesench/neural) (see the accompanying ReactomePA plot in Supplementary figure 9.

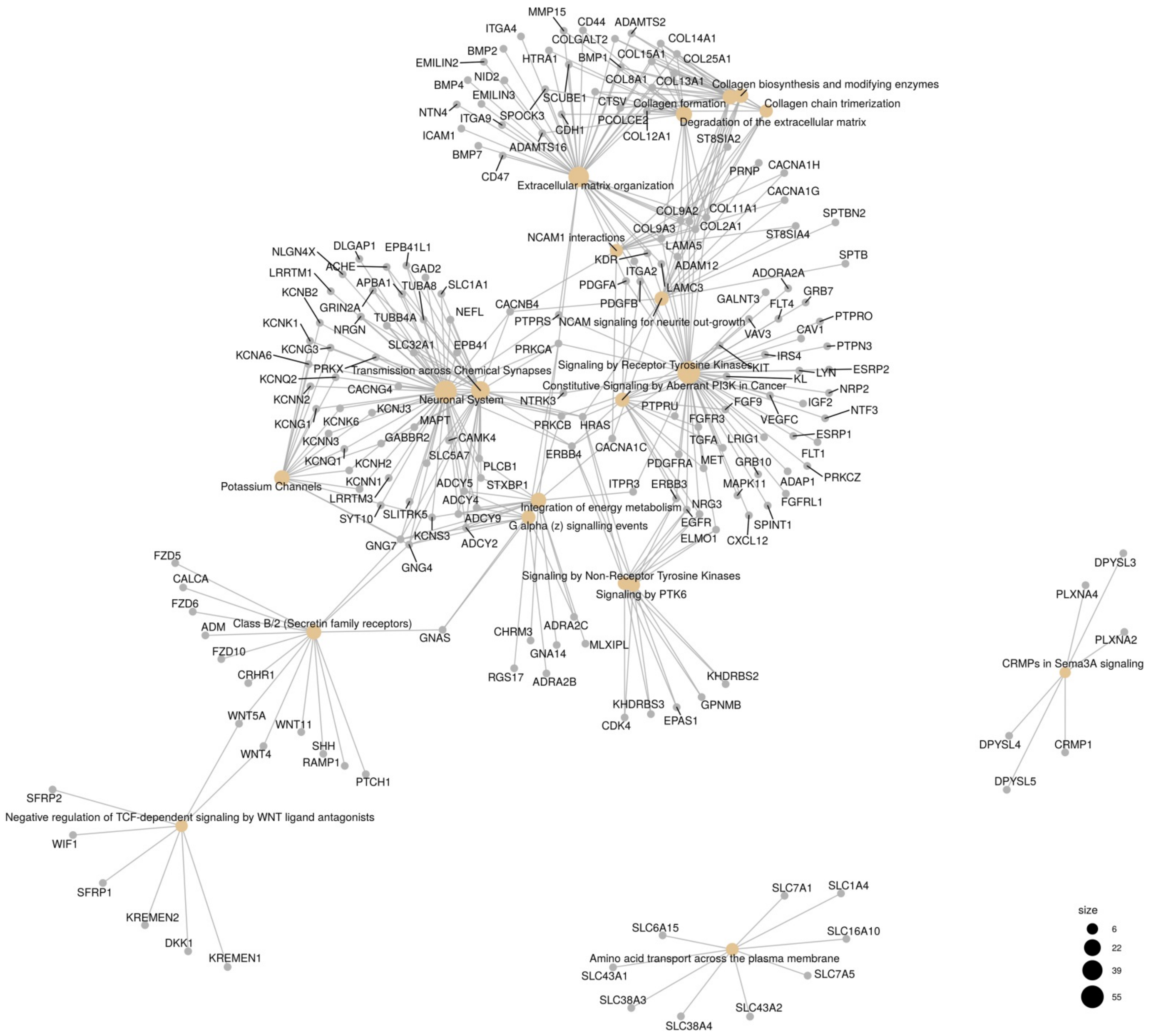

Supplementary figure 9. ReactomePA plot for genes underlying the dual H3K27me3 and H3K4me3 signal in kidney.

This figure relates to Supplementary figure 8. The analysis shows gene set enrichment and functional analysis as part of ReactomePA[2]. The visualisation, which can be magnified, demonstrates the major functional annotations for the dual H3K27me3 and H3K4me3 signal in Supplementary figure 8 relate to common features of mesenchyme/vascular (e.g. ECM organisation, collagens, and receptor tyrosine kinase signalling) and neural tissue (e.g. neuronal system, transmission across chemical synapses and neurite outgrowth). These findings were common to the sub-category of dual H3K27me3 and H3K4me3 signal in all tissues. Circle size reflects the number of connected genes.

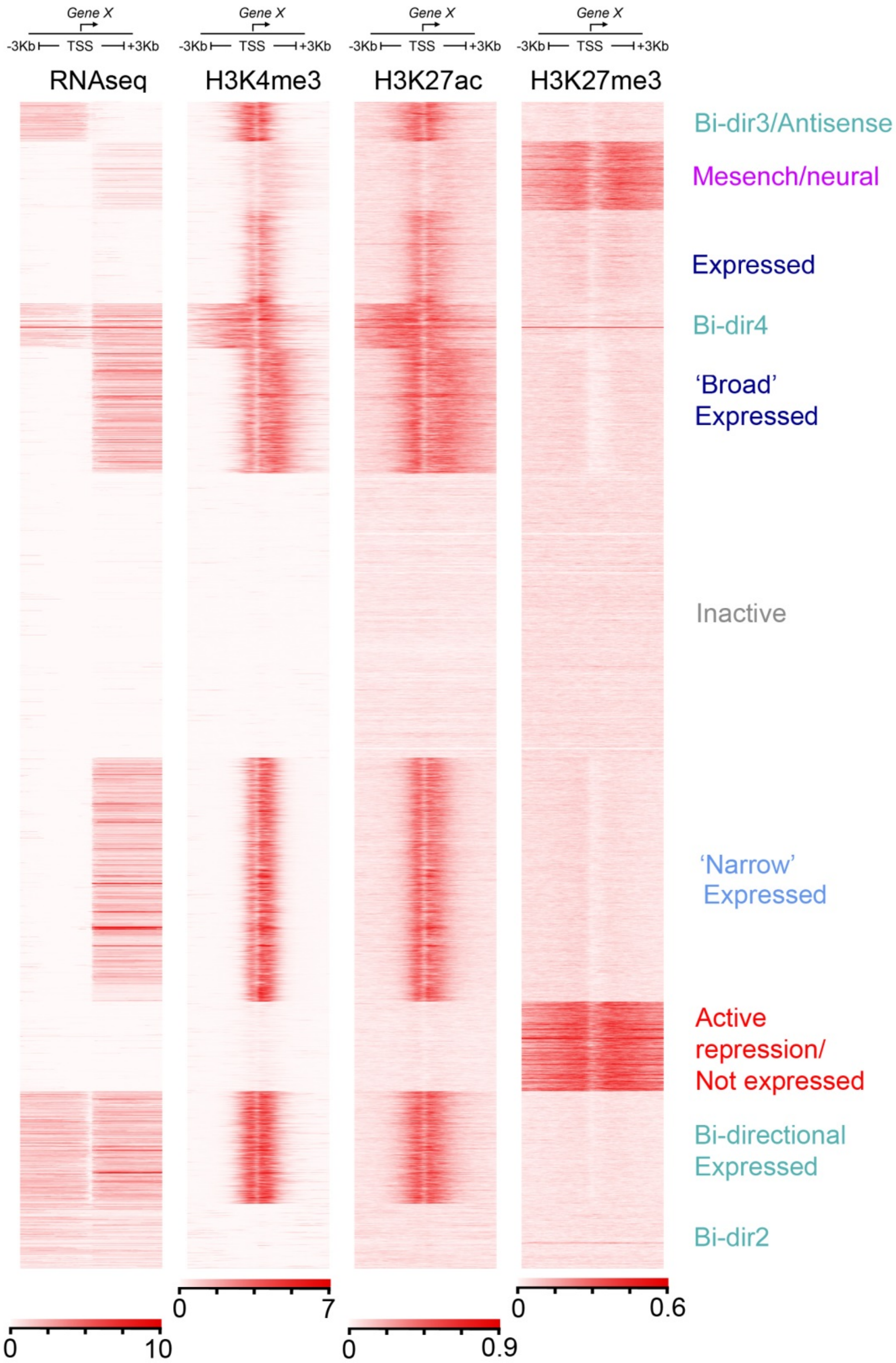

Supplementary figure 10. Extended categorisation of promoter states in pancreas to identify a combined H3K27me3 / H3K4me3 signal.

This example is for pancreas, which required extension of ngsplot parameters[1] to allow ten clusters in order to split the H3K27me3 signal and identify a sub-category which also contained H3K4me3 (as a by-product, this modification also identified the major promoter states identified in Supplementary figures 2 & 3 and additional sub-categories of bidirectional or antisense transcripts). Rather than bivalency, the genes underlying the combined H3K27me3 and H3K4me3 signal were characteristic of mesenchyme/vascular and nerves (Mesench/neural) (see the accompanying ReactomePA plot in Supplementary figure 11.

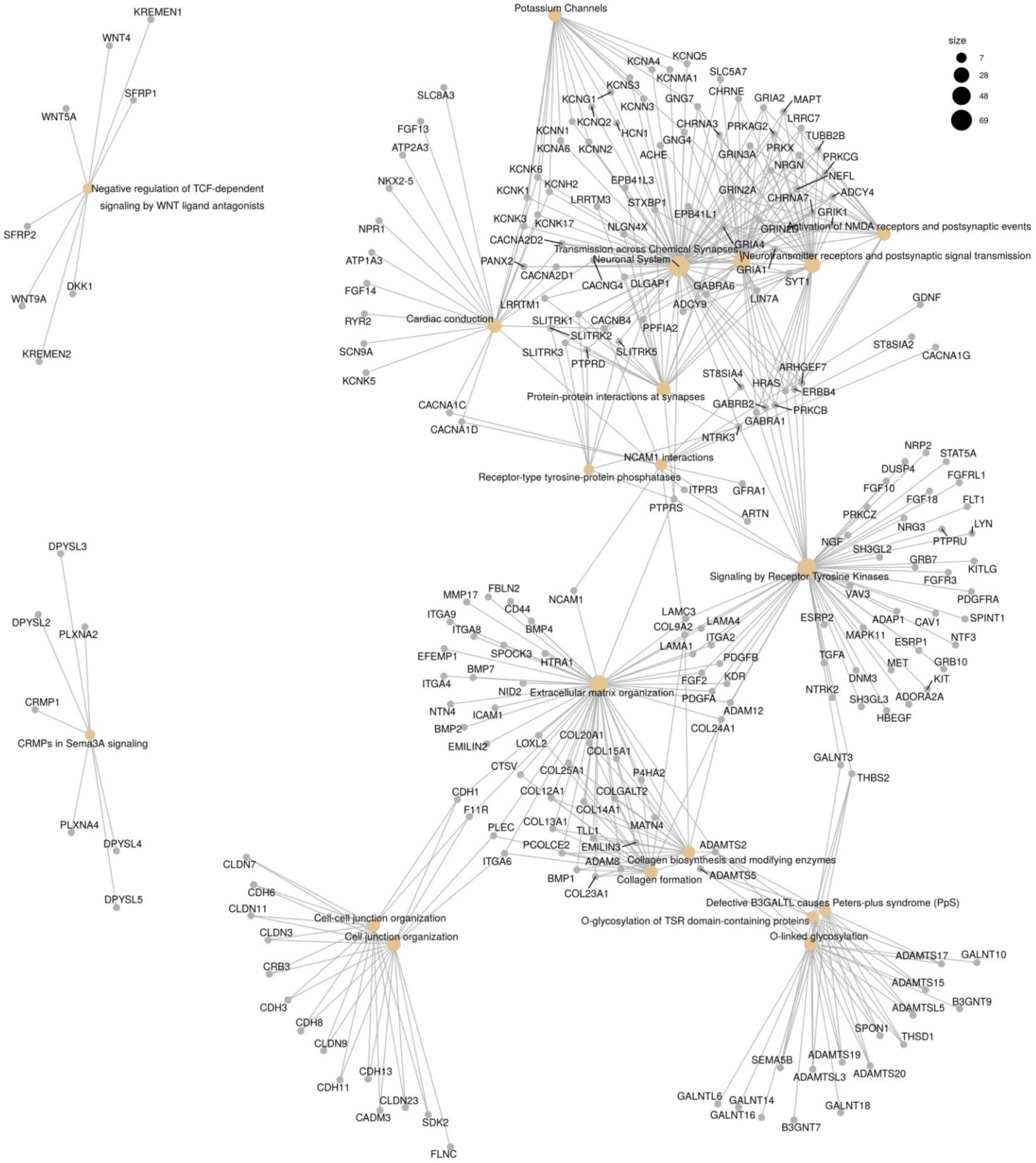

Supplementary figure 11. ReactomePA plot for genes underlying the dual H3K27me3 and H3K4me3 signal in pancreas.

This figure relates to Supplementary figure 10. The analysis shows gene set enrichment and functional analysis as part of ReactomePA[2]. The visualisation, which can be magnified, demonstrates the major functional annotations for the dual H3K27me3 and H3K4me3 signal in Supplementary figure 10 relate to common features of mesenchyme/vascular (e.g. ECM organisation, collagens, and tyrosine kinase signalling) and neural tissue (e.g. neuronal system and transmission across chemical synapses). These findings were common to the sub-category of dual H3K27me3 and H3K4me3 signal in all tissues. Circle size reflects the number of connected genes.

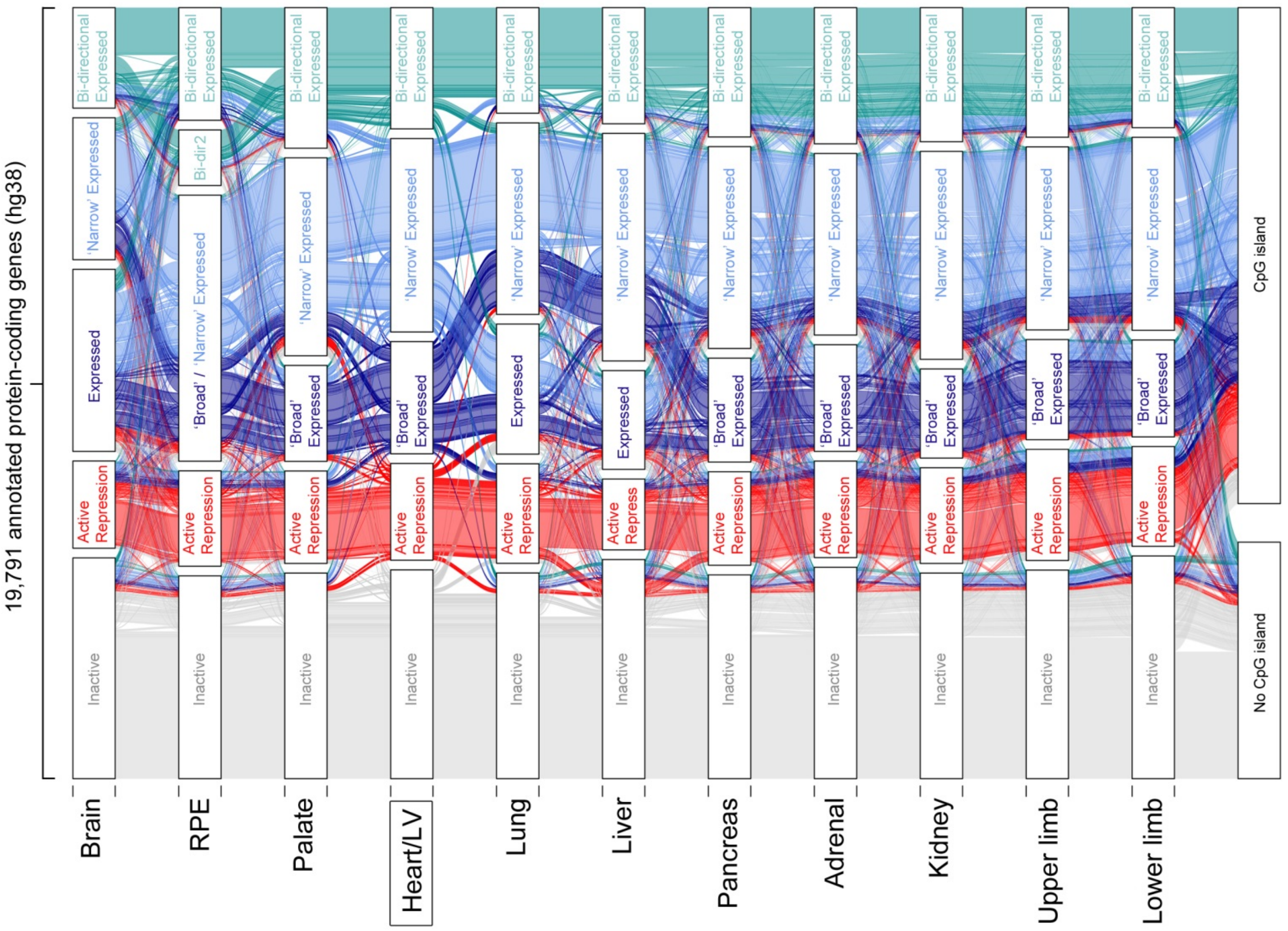

Supplementary figure 12. Integration of all identified promoter states across tissues.

This figure relates to Figure 3. Alluvial plot showing promoter state for 19,791 annotated genes across all tissues with replicated datasets. All the amalgamated promoter states associated with gene transcription in Figure 3 are shown individually here: Broad expressed, Narrow expressed, Expressed, Bidir and Bidir2. The plot shown is centred on (and with variance from) the promoter state in the Heart/LV dataset. The plot also categorises genes according to the presence or absence of a CpG island at the promoter. Genes with an Inactive promoter state characteristically lacked a CpG island. Promoters either actively transcribed or repressed tended to possess a CpG island.

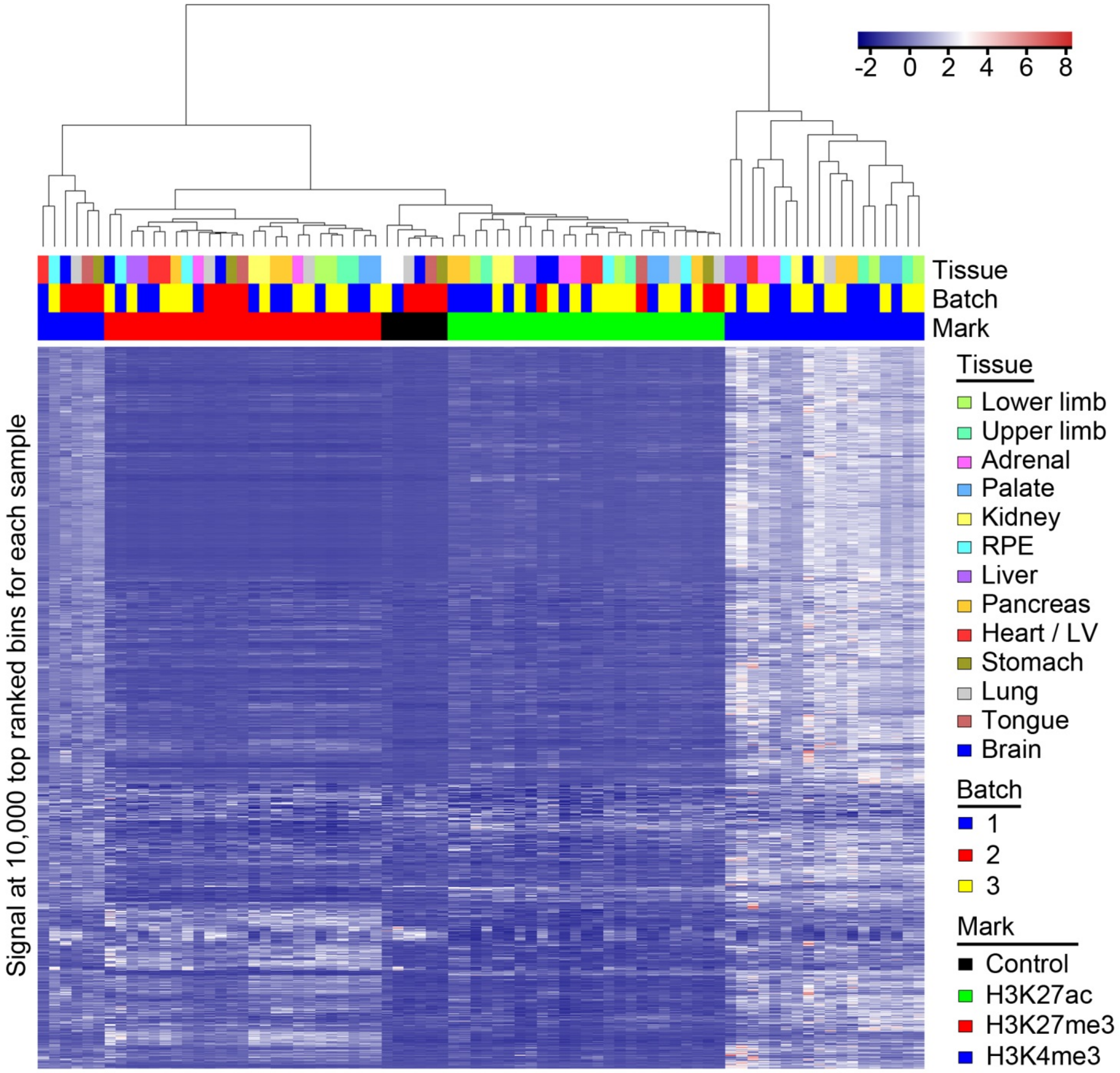

Supplementary figure 13. Heatmap showing hierarchical clustering of ChIPseq datasets. Heatmap showing hierarchical clustering of ChIPseq datasets based on the combined set of 10,000 most highly ranked bins from each sample. Samples clustered according to mark and did not cluster according to sequencing batch.

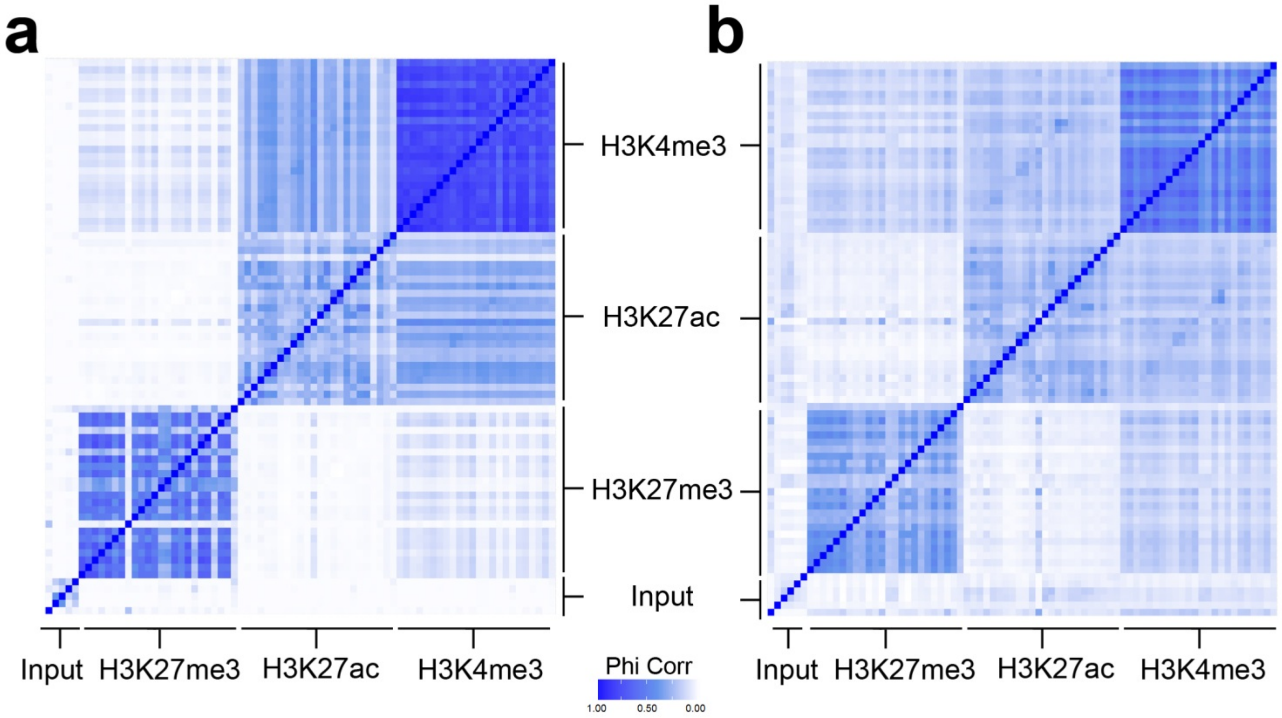

Supplementary figure 14. Heatmap showing Phi correlation between samples when peaks are called by MACs or allocated according to read count into 1 kb bins.

a) Peak calling by MACs[3]. b) Allocation according to read counts into 1 kb bins. The two approaches produced similar heatmaps thus benchmarking the 1 kb bin approach. Each histone modification is comprised of 26 individual rows and columns for the 12 tissue replicates plus single datasets for tongue and stomach. The input is comprised of 5 control samples. The dark blue diagonal line is the perfect correlation from assessing each sample against itself.

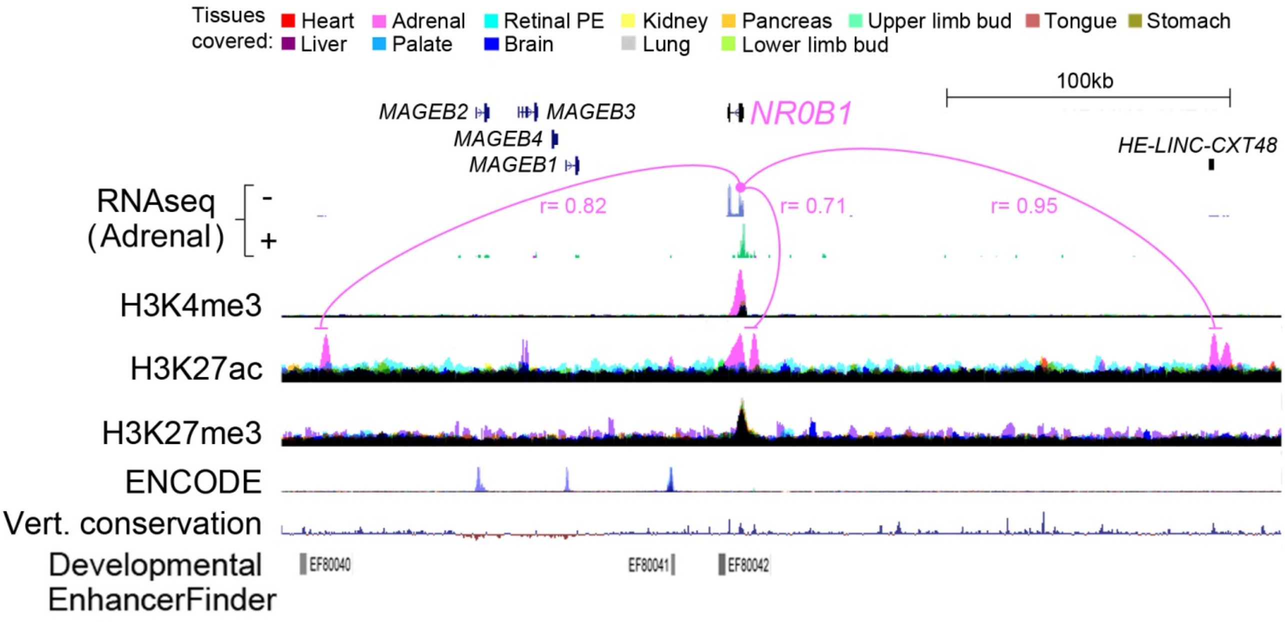

Supplementary figure 15. Adrenal-specific epigenomic landscape over 300 kb at the *NR0B1* locus on the X chromosome.

Assembled tracks show RNAseq for the adrenal and multi-layered data for all tissues for each histone modification. One replicate of each track is shown for simplicity. *NR0B1* was only expressed in the adrenal and has an adrenal-specific H3K4me3 mark. Multiple adrenal-specific H3K27ac enhancer peaks were visible across 300 kb, all highly correlated with the expression of *NR0B1*. Some of the enhancers are poorly conserved, unpredicted by in silico tools (Developmental Enhancer Finder[4]), and absent from ENCODE datasets[5]. Identifying multiple enhancers facilitates their grouping to assist with statistical power when assessing the potential pathogenicity of patient variants in whole genome sequencing data.

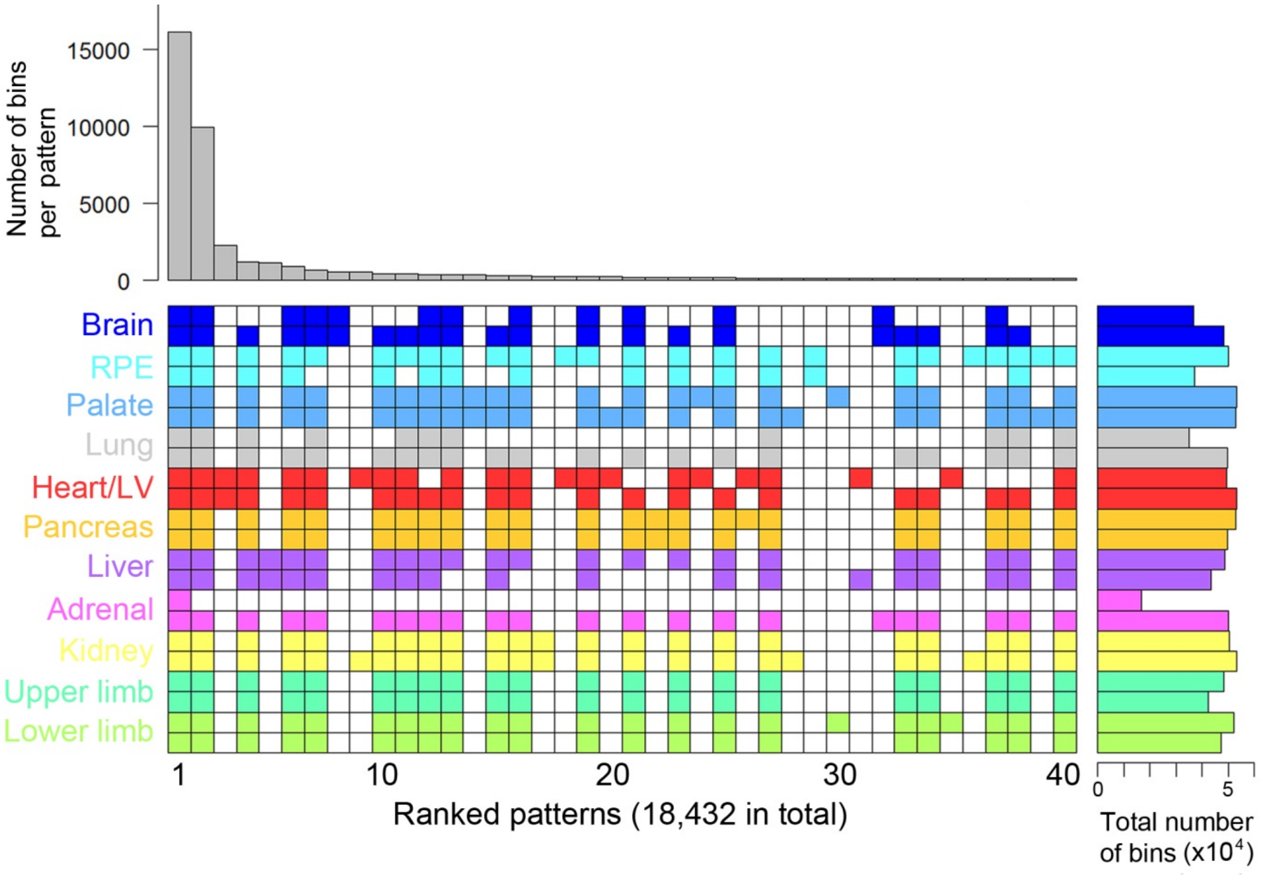

Supplementary figure 16. Patterns of H3K4me3 across human embryonic tissues with the requirement of detection in at least 2 samples.

This figure relates to Figure 5. Euler grid for bins marked by H3K4me3 (defined by elbow plots) in replicated tissues (i.e. two rows/replicates per tissue). Total number of marked bins per individual dataset is shown to the right. The grid shown required a bin to be called in any two or more samples and is ordered by decreasing bin count per pattern (bar chart above the grid). A total of 18,432 different patterns were identified (far fewer that the 48,570 found for the corresponding analysis of H3K27ac). The top 40 are shown. All tissue-specific patterns emerged in the top 2,267 (within the top 12.3% of patterns).

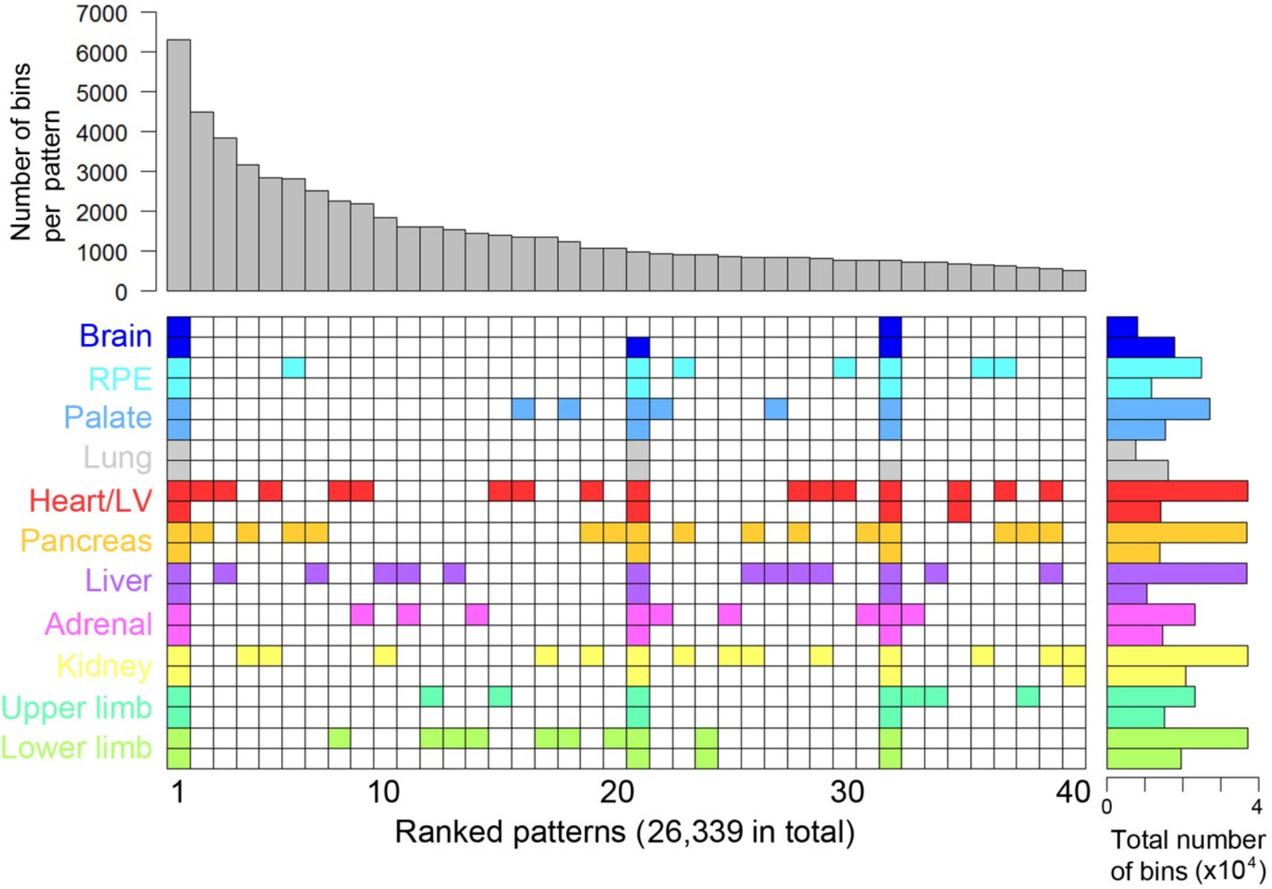

Supplementary figure 17. Patterns of H3K27me3 across human embryonic tissues with the requirement of detection in at least 2 samples.

This figure relates to Figure 5. Euler grid for bins marked by H3K27me3 (defined by elbow plots) in replicated tissues (i.e. two rows/replicates per tissue). Total number of marked bins per individual dataset is shown to the right. The grid shown required a bin to be called in any two or more samples and is ordered by decreasing bin count per pattern (bar chart above the grid). A total of 26,339 different patterns were identified (far fewer that the 48,570 found for the corresponding analysis of H3K27ac). The top 40 are shown. All tissue-specific patterns emerged in the top 836 (within the top 3.2% of patterns).

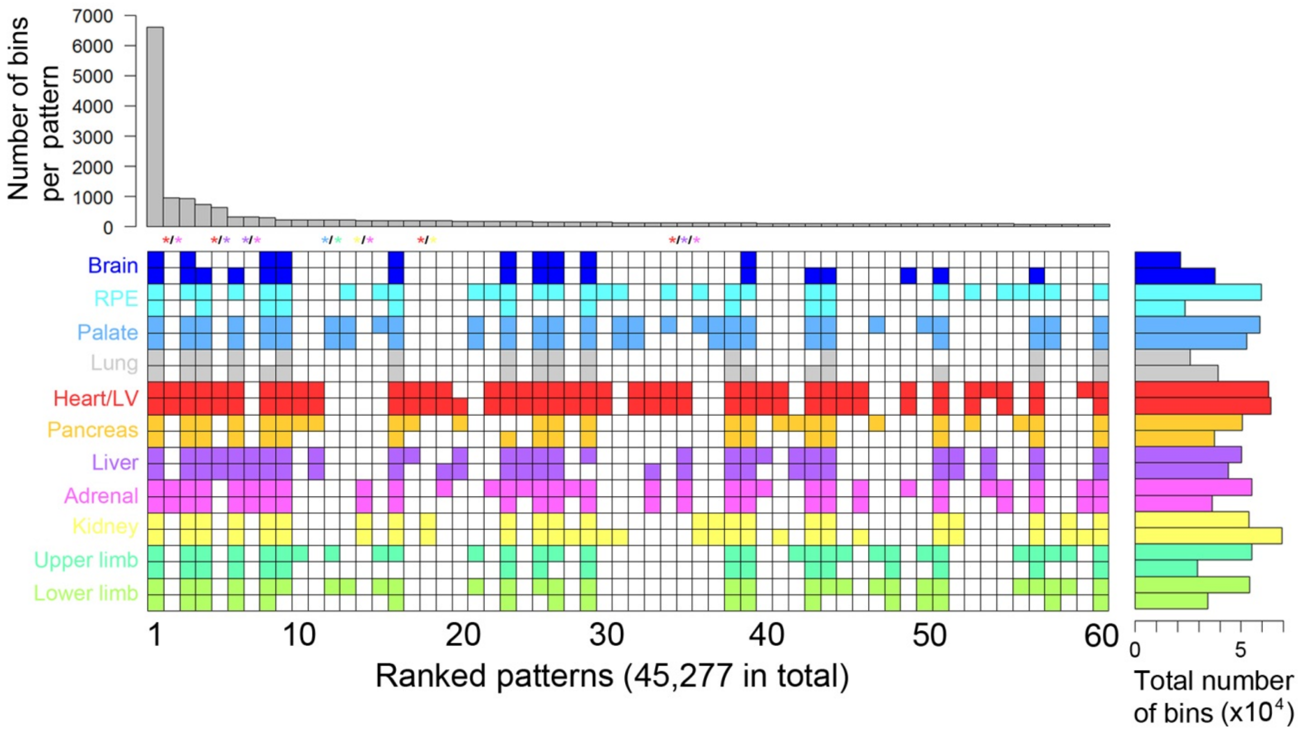

Supplementary figure 18. Patterns of H3K27ac across tissues with the requirement of detection in at least 4 samples.

This figure relates to Figure 5. Euler grid is shown for bins marked by H3K27ac (defined by elbow plots). Detecting patterns shared across tissues was enforced by detection in at least four samples (which must include at least two tissues). The grid includes replicated tissues (i.e. two rows/replicates per tissue). Total number of marked bins per individual dataset is shown to the right. The grid is ordered by decreasing bin count per pattern (bar chart above the grid). A total of 45,277 different patterns were identified. The top 60 are shown. Colour-coded asterisks above the columns indicate patterns shared uniquely across two (heart-adrenal, heart-liver, palate-limb, kidney-adrenal and heart-kidney) or three (heart-liver-adrenal) tissues.

SUPPLEMENTARY REFERENCES

1. Shen, L., Shao, N., Liu, X. & Nestler, E. ngs.plot: Quick mining and visualization of next-generation sequencing data by integrating genomic databases. *BMC Genomics* **15**, 284 (2014).
2. Yu, G. & He, Q.Y. ReactomePA: an R/Bioconductor package for reactome pathway analysis and visualization. *Mol Biosyst* **12**, 477-479 (2016).
3. Zhang, Y., Liu, T., Meyer, C.A., Eeckhoute, J., Johnson, D.S., Bernstein, B.E., Nusbaum, C., Myers, R.M., Brown, M., Li, W. & Liu, X.S. Model-based analysis of ChIP-Seq (MACS). *Genome biology* **9**, R137+ (2008).
4. Erwin, G.D., Oksenberg, N., Truty, R.M., Kostka, D., Murphy, K.K., Ahituv, N., Pollard, K.S. & Capra, J.A. Integrating diverse datasets improves developmental enhancer prediction. *PLoS Comput Biol* **10**, e1003677 (2014).
5. Ernst, J. et al. Mapping and analysis of chromatin state dynamics in nine human cell types. *Nature* **473**, 43-49 (2011).
