# Supplementary material for: Dynamic changes in the epigenomic landscape regulate human organogenesis and link to developmental disorders
Source: Nat Commun. 2020 Aug 6;11:3920. doi: 10.1038/s41467-020-17305-2 (PMC7413392; doi:10.1038/s41467-020-17305-2)
Supplement: Supplementary file 12 — Reporting Summary [file 41467_2020_17305_MOESM12_ESM.pdf]

nature research

Corresponding author(s): Neil Hanley

Last updated by author(s): May 29, 2020

Reporting Summary

Nature Research wishes to improve the reproducibility of the work that we publish. This form provides structure for consistency and transparency in reporting. For further information on Nature Research policies, seeAuthors & Referees and theEditorial Policy Checklist .

Statistics

For all statistical analyses, confirm that the following items are present in the figure legend, table legend, main text, or Methods section.

n/a	Confirmed	
☐	☒	The exact sample size (*n*) for each experimental group/condition, given as a discrete number and unit of measurement
☐	☒	A statement on whether measurements were taken from distinct samples or whether the same sample was measured repeatedly
☐	☒	The statistical test(s) used AND whether they are one- or two-sided<br>*Only common tests should be described solely by name; describe more complex techniques in the Methods section.*
☒	☐	A description of all covariates tested
☐	☒	A description of any assumptions or corrections, such as tests of normality and adjustment for multiple comparisons
☐	☒	A full description of the statistical parameters including central tendency (e.g. means) or other basic estimates (e.g. regression coefficient) AND variation (e.g. standard deviation) or associated estimates of uncertainty (e.g. confidence intervals)
☐	☒	For null hypothesis testing, the test statistic (e.g. *F*, *t*, *r*) with confidence intervals, effect sizes, degrees of freedom and *P* value noted<br>*Give P values as exact values whenever suitable.*
☐	☒	For Bayesian analysis, information on the choice of priors and Markov chain Monte Carlo settings
☒	☐	For hierarchical and complex designs, identification of the appropriate level for tests and full reporting of outcomes
☐	☒	Estimates of effect sizes (e.g. Cohen's *d*, Pearson's *r*), indicating how they were calculated

Our web collection on statistics for biologists contains articles on many of the points above.

Software and code

Policy information about availability of computer code

Data collection	We have provided these descriptions in the Methods including the version and referenced the source. The below is a list of packages and versions.<br>Bowtie 1.0.0<br>MACS2 2.0.10.20131216<br>STAR 2.4.2a<br>chromHMM 1.11<br>ngs.plot 2.61<br>Alluvial Diagrams 0.2-0<br>xEnricherGenes of XGR 1.1.1<br>ReactomePA 3.10<br>csaw 3.11<br>subseq 1.0.0<br>plotEuler / Biostars 6.1.0<br>HOMER v4.9<br>CHOPCHOP version 3
Data analysis	Please also see preceding section. We have provided these descriptions in the Methods including the version and referenced the source. This includes new code freely available on Github and fully referenced with hyperlink in the manuscript.<br><br>Imaging software used in the study:<br>CellSens Olympus software<br>Fiji ImageJ

New codes available from:
arseFromElbow. https://rdrr.io/github/davetgerrard/utilsGerrardDT/src/R/arseFromElbow.R
plotEuler. https://github.com/davetgerrard/utilsGerrardDT

For manuscripts utilizing custom algorithms or software that are central to the research but not yet described in published literature, software must be made available to editors/reviewers. We strongly encourage code deposition in a community repository (e.g. GitHub). See the Nature Research guidelines for submitting code & software for further information.

Data

Policy information about availability of data

All manuscripts must include a data availability statement. This statement should provide the following information, where applicable:

- Accession codes, unique identifiers, or web links for publicly available datasets
- A list of figures that have associated raw data
- A description of any restrictions on data availability

ChIPseq and RNAseq datasets have been deposited in the European Genome Phenome repository (https://www.ebi.ac.uk/ega/home) under accessions: EGAS00001003738 and EGAS00001003163. Supplementary tables 1-3 detail the human embryonic material contributing to these datasets. To view data in the UCSC genome browser, a trackhub is at http://www.humandevelopmentalbiology.manchester.ac.uk/.
The following databases were used by the study:
ENCODE (https://www.encodeproject.org/)
NIH Roadmap (http://www.roadmapepigenomics.org/)
FANTOM5 (https://fantom.gsc.riken.jp/5/)
xEnricherGenes (from XGR v1.1.1)
Reactome pathway database (https://reactome.org/)
HOMER v4.9 (http://homer.ucsd.edu/homer/)

Field-specific reporting

Please select the one below that is the best fit for your research. If you are not sure, read the appropriate sections before making your selection.

☒ Life sciences ☐ Behavioural & social sciences ☐ Ecological, evolutionary & environmental sciences

For a reference copy of the document with all sections, see nature.com/documents/nr-reporting-summary-flat.pdf

Life sciences study design

All studies must disclose on these points even when the disclosure is negative.

Sample size

No sample size was chosen for the human embryo collection that contributed to the study. For the zebrafish transgenic experiments we ensured multiple stable transgenic founder lines were established (range: 3 to 7 according to the usual variation in the success of trangenesis). All founder lines were included in subsequent analyses of reporter gene expression. 100% of founder lines yielded correct reporter gene expression strongly implying that sample sizes were sufficient. These details are included in Supplementary table 6 and referred to in the manuscript text.
For the example analysis of wildtype and mutant enhancer cardiomyocyte differentiation of hPSCs, a power calculation was not undertaken because it was not possible at the outset to infer an effect size. Therefore, we ensured an analysis of a large number of embryoid bodies, aiming at 30 per group. The control group was reduced to 29 because of one technical failure of cell culture. All EBs were included in the analyses of GFP fluorescence and gene expression across three independent experiments. Conclusive results were obtained for RBM24 gene expression.

Data exclusions

The only data excluded are described in the Methods for the 1kb binning which pertains to the results from Figure 5 onwards: 'Reads from mitochondrial and unplaced chromosome annotations were removed. A further 697 bins were filtered out for possessing >10,000 reads in all samples or if the mean read count from input controls was >50% of the mean read count of all samples or for being situated in pericentromeric regions (using table ideogram from UCSC; listed in Supplementary table 8).' Mitochondrial reads were excluded because they emanate from a separate genome. Annotations that were unplaced were excluded because there is no reliability about their origin. The 697 bins with massively high read counts across all samples were excluded because of the near certainty of these reads being technical artifacts. These exclusion criteria were pre-established as part of commonplace analysis pipelines for ChIPseq data.

Replication

All results were reproducible. For the ChIPseq data replicates were undertaken for 11 out of the 13 tissues. Where investigation is restricted to the replicated samples this is clearly stated in the manuscript Results text. For the promoter state analysis, both replicates gave equivalent results and the same categorization. For the zebrafish trangenics, the expected profile of GFP detection was observed in 100% of multiple founder lines. Major batch effect was excluded by hierarchical clustering (Supplementary figure 13). Correlation was proven between MACS peak calling and the 1kb binning approach and shown in Supplementary figure 14. Replication was ensured for the hPSC differentiation by undertaking independent experiments in triplicate with each group containing 10 embryoid bodies (except one control group with 9).

Randomization

This section on Randomzation does not feel relevant to the approaches that we undertook which are described in the manuscript. Analysis was undertaken on specific tissues with no element of different treatments being undertaken (e.g. as would need randomization to avoid bias in drug treatment trials).

Blinding	Blinding is not relevant to the undertaking of the RNAseq and ChIPseq analyses because the genome-scale bioinformatic investigations are not user-defined, i.e. the opportunity for user bias is removed. For the zebrafish analysis the images are provided as the data. While blinding would theoretically be possible for the analysis of fluroescence in the wildtype and mutant embryoid bodies, in reality the entire EB was subject to automated fluorescent measurement in providing the data in Fig. 8e, such that user bias would not be possible.

Reporting for specific materials, systems and methods

We require information from authors about some types of materials, experimental systems and methods used in many studies. Here, indicate whether each material, system or method listed is relevant to your study. If you are not sure if a list item applies to your research, read the appropriate section before selecting a response.

Materials & experimental systems

n/a	Involved in the study
☐	☒ Antibodies
☒	☐ Eukaryotic cell lines
☒	☐ Palaeontology
☐	☒ Animals and other organisms
☐	☒ Human research participants
☒	☐ Clinical data

Methods

n/a	Involved in the study
☐	☒ ChIP-seq
☒	☐ Flow cytometry
☒	☐ MRI-based neuroimaging

Antibodies

Antibodies used	The antibodies used are described in the 'ChIPseq antibody section' below.
Validation	These antibodies have been extensively used for ChIP-seq histone modification analyses.

Animals and other organisms

Policy information about studies involving animals; ARRIVE guidelines recommended for reporting animal research

Laboratory animals	Zebrafish (danio rerio), embryos are injected prior to knowing sex at the 1-cell stage.
Wild animals	No wild animals were used
Field-collected samples	No field collected samples were used
Ethics oversight	All protocols used have been approved by the Ethics Committee of the Andalusian Government (license numbers 450-1839 and 182-41106 for CABD-CSIC-UPO). This is stated in the Methods.

Note that full information on the approval of the study protocol must also be provided in the manuscript.

Human research participants

Policy information about studies involving human research participants

Population characteristics	Embryonic material was collected from women undergoing termination of pregnancy in Manchester University NHS Foundation Trust. The women referred to this clinical service represent the diverse ethnicity and demographics of women of fertile age (over the age of 16) in the Greater Manchester region of the UK.
Recruitment	We approach all women who have given clinical consent within the confines of our ethical approval (over 16 years, without undue emotional distress). Our study population reflects the ethnically diverse population of Greater Manchester and so ascertainment bias is not anticipated compared to other human embryonic tissue sources.
Ethics oversight	North West Regional Ethics Committee as stated in the manuscript.

Note that full information on the approval of the study protocol must also be provided in the manuscript.

ChIP-seq

Data deposition

☒ Confirm that both raw and final processed data have been deposited in a public database such as GEO.

☒ Confirm that you have deposited or provided access to graph files (e.g. BED files) for the called peaks.

Data access links<br>*May remain private before publication.*	ChIPseq and RNAseq datasets have been deposited in the European Genome Phenome repository under accessions: EGAS00001003738 and EGAS00001003163. Supplementary tables 1-3 detail the human embryonic material contributing to

these datasets. To view data in the UCSC genome browser, a trackhub is at http://www.humandevelopmentalbiology.manchester.ac.uk/. Codes are freely available under references 55 and 56.

Files in database submission

These are extensive and detailed in Supplementary tables 1-3 including unique cross-referencing codes. The RNAseq datasets are pasted here:
Batch Sample_id Source/novel Database accession
BATCH1 Adrenal_1 Gerrard et al., 2016 eLife E-MTAB-3928
BATCH2 Adrenal_2 Gerrard et al., 2016 eLife E-MTAB-3928
BATCH3 Brain_1 Gerrard et al., 2016 eLife E-MTAB-3928
BATCH3 Brain_2 Gerrard et al., 2016 eLife E-MTAB-3928
BATCH1 Kidney_1 Gerrard et al., 2016 eLife E-MTAB-3928
BATCH2 Kidney_2 Gerrard et al., 2016 eLife E-MTAB-3928
BATCH1 Liver_1 Gerrard et al., 2016 eLife E-MTAB-3928
BATCH2 Liver_2 Gerrard et al., 2016 eLife E-MTAB-3928
BATCH2 Lower_limb_2 Gerrard et al., 2016 eLife E-MTAB-3928
BATCH3 Lower_limb_3 Gerrard et al., 2016 eLife E-MTAB-3928
BATCH3 Lung_1 Gerrard et al., 2016 eLife E-MTAB-3928
BATCH3 Lung_2 Gerrard et al., 2016 eLife E-MTAB-3928
BATCH1 Palate_1 Gerrard et al., 2016 eLife E-MTAB-3928
BATCH2 Palate_2 Gerrard et al., 2016 eLife E-MTAB-3928
BATCH0 Pancreas_1 Cebola et al., 2015 Nat Cell Biol E-MTAB-3061
BATCH4 Pancreas_2 New TBD
BATCH1 RPE_1 Gerrard et al., 2016 eLife E-MTAB-3928
BATCH2 RPE_2 Gerrard et al., 2016 eLife E-MTAB-3928
BATCH3 Stomach_1 Gerrard et al., 2016 eLife E-MTAB-3928
BATCH3 Stomach_2 Gerrard et al., 2016 eLife E-MTAB-3928
BATCH3 Tongue_1 Gerrard et al., 2016 eLife E-MTAB-3928
BATCH4 Tongue_2 New TBD
BATCH2 Upper_limb_2 Gerrard et al., 2016 eLife E-MTAB-3928
BATCH3 Upper_limb_3 Gerrard et al., 2016 eLife E-MTAB-3928
BATCH1 Ventricle_1 Gerrard et al., 2016 eLife E-MTAB-3928
BATCH2 Ventricle_2 Gerrard et al., 2016 eLife E-MTAB-3928

Genome browser session
(e.g. UCSC)

To view data in the UCSC genome browser, a trackhub is at http://www.humandevelopmentalbiology.manchester.ac.uk/.

Methodology

Replicates

All datasets contained ChIPseq replicates except for stomach and tongue as described in the manuscript and explcuded from replicated analyses.

Sequencing depth

There are very many files not suitalbe for this form. They are fully listed in Suppplementary table 2.

Antibodies

Antibody Company Catalog number
Anti-H3K4me3 Millipore 05-745R
Anti-H3K27ac Abcam AB4729
Anti-H3K27me3 Millipore 07-449

Peak calling parameters

There is a section of the Methods on this:
The first batch of ChIPseq was mapped originally to hg19 using Bowtie 1.0.0 (parameters -m1 -n2 -l28, uniquely mapped reads only)43 and peaks called using MACS2 (2.0.10.20131216)44 against a common input sample (derived from all tissues). MACS parameters used were as follows: band-width 300 bp, mfold 5 to 50 (used in cross-correlation for fragment length estimation), q-value cut off 0.05. To prioritise candidate enhancers for transgenic testing, H3K27ac data from ENCODE (7 cell lines) and NIH Roadmap (154 samples)10,26 were mapped similarly. Subsequently, all data, including the external H1 hPSC and adult pancreas data (Figure 3c), were mapped to hg38 using STAR (2.4.2a)45. ChIPseq reads were trimmed to 50 bp for consistency and only uniquely mapped reads were retained. For ChIPseq, spliced mappings were suppressed by setting the parameter "alignIntronMax" to 1. The full STAR parameters for ChIPseq were as follows: " --alignIntronMax 1, --seedSearchStartLmax 30, --outSAMattributes All, and --outSAMtype BAM SortedByCoordinate". GENCODE 25 gene annotations were used for RNAseq mapping and read counting46. The full STAR parameters for RNA-seq were as follows: "—outSAMattributes All, --quantMode GeneCounts –out, SAMtype BAM SortedByCoordinate".

Data quality

As described in full in the manuscript, we undertook comparative analyses between our peak calling and our 1kb binning approach. This included Phi correlation and hierarchical clustering both of which are included as Supplementary figures 13-14.

Software

From the Methods (numbers in parentheses indicate the corresponding reference):
Bowtie 1.0.0 (parameters -m1 -n2 -l28, uniquely mapped reads only) (43) and peaks called using MACS2 (2.0.10.20131216) (44) against a common input sample (derived from all tissues).
Genomic segmentation was performed using chromHMM (version 1.11) (17)
Lists of genes from the associated promoter state were tested for enrichment of annotations using the xEnricherGenes

function from the R package XGR version 1.1.1 under default parameters (48).
Pathway annotation and gene set enrichment analysis for the subset of H3K27me3/H3K4me3 dual marked promoters in each tissue was undertaken using ReactomePA (release 3.10) under default parameters, which associates genes to their known functions based on the REACTOME pathway database (49).
For 1 kb bins, reads were counted into bins according to their mapped start position using csaw (53).
For Pearson correlations with surrounding transcription binned read counts were down-sampled statistically using subSeq (54) weighting each sample by the value of the 99th percentile.
arseFromElbow to determine elbos plot thresholds in 1kb binning from a vector of counts is available on github (55).
HOMER v4.9 was used to search for enriched motifs in selected sets of bins (59).
