# Supplementary material for: Dynamic changes in the epigenomic landscape regulate human organogenesis and link to developmental disorders
Source: Nat Commun. 2020 Aug 6;11:3920. doi: 10.1038/s41467-020-17305-2 (PMC7413392; doi:10.1038/s41467-020-17305-2)
Supplement: Supplementary file 13 — Description of Additional Supplementary Files [file 41467_2020_17305_MOESM13_ESM.pdf]

Title: Supplementary Data 1.
Description: Human embryonic tissue samples contributing to the study.
Human embryonic tissues are coded with a unique identifier to allow tracking across different datasets. M, male; F, female.

Title: Supplementary Data 2.
Description: ChIPseq statistics and antibodies.
ChIPseq datasets generated in this study and their associated metadata. Antibodies used against the histone modification and their source.

Title: Supplementary Data 3.
Description: RNAseq datasets used in this study.
Gerrard et al., 2016 is Ref. 15 in the manuscript. Cebola et al., 2015 is Ref. 13 in the manuscript.

Title: Supplementary Data 4.
Description: Promoter states for all annotated genes in replicated tissues.
The column 'CpG.1kb' denotes transcripts with a start site within 1 kb of an annotated CpG island (UCSC hg38 database). The small subset of NAs in this column (297 out of 19,791) represent transcripts in the ngsplt hg38 protein-coding set that are not referenced in the GENCODE25 annotation set.

Title: Supplementary Data 5.
Description: Gene sets that are Expressed or Disallowed according to tissue or over time
a) Gene sets with an Expressed promoter state in a replicated organ/tissue and 'Active repression' (disallowed) promoter state in other tissues. b) Gene sets with an Expressed promoter state in either embryonic pancreas (Row 16) or adult pancreas (Row 17, i.e. the second named organ) and 'Active repression' (disallowed) promoter state in first named cell population/organ.
These data link to Figure 3b of the main manuscript.

Title: Supplementary Data 6.
Description: Zebrafish transgenics.
Locations are shown in hg38. *For the enhancer 141 kb from *ALX1*, 7/7 founders gave appropriate trabecular expression while 5/7 resulted in mandibular reporter gene activity. Hpf, hours post-fertilisation.

Title: Supplementary Data 7.
Description: Ranking of tissue specificity for 1 kb bins marked with H3K27ac for all replicated organs and tissues.
Tissue specificity ranked in the top 0.6% of patterns with most organs ranking much higher. These data point to high consistency of tissue samples and experimental processes between replicates.

Title: Supplementary Data 8.
Description: Excluded 1 kb bins.

Title: Supplementary Data 9.
Description: Realtime PCR primers and guide RNAs.
